# Oral Delivery of DMAB-Modified Docetaxel-Loaded PLGA-TPGS Nanoparticles for Cancer Chemotherapy

**DOI:** 10.1007/s11671-010-9741-8

**Published:** 2010-08-20

**Authors:** Hongbo Chen, Yi Zheng, Ge Tian, Yan Tian, Xiaowei Zeng, Gan Liu, Kexin Liu, Lei Li, Zhen Li, Lin Mei, Laiqiang Huang

**Affiliations:** 1 School of Life Sciences, Tsinghua University, 100084, Beijing, People's Republic of China; 2 The Shenzhen Key Lab of Gene and Antibody Therapy, Center for Biotech and Bio-Medicine and Division of Life Sciences, Graduate School at Shenzhen, Tsinghua University, Shenzhen, Guangdong Province 518055, China; 3 College of Pharmacy, Dalian Medical University, 116027, Dalian Liaoning, People's Republic of China; 4 Key Laboratory of Functional Polymer Materials, Ministry of Education; Institute of Polymer Chemistry, Nankai University, 300071, Tianjin, People's Republic of China

**Keywords:** PLGA-TPGS, DMAB, Nanoparticle, Oral Chemotherapy, Docetaxel

## Abstract

Three types of nanoparticle formulation from biodegradable PLGA-TPGS random copolymer were developed in this research for oral administration of anticancer drugs, which include DMAB-modified PLGA nanoparticles, unmodified PLGA-TPGS nanoparticles and DMAB-modified PLGA-TPGS nanoparticles. Firstly, the PLGA-TPGS random copolymer was synthesized and characterized. DMAB was used to increase retention time at the cell surface, thus increasing the chances of particle uptake and improving oral drug bioavailability. Nanoparticles were found to be of spherical shape with an average particle diameter of around 250 nm. The surface charge of PLGA-TPGS nanoparticles was changed to positive after DMAB modification. The results also showed that the DMAB-modified PLGA-TPGS nanoparticles have significantly higher level of the cellular uptake than that of DMAB-modified PLGA nanoparticles and unmodified PLGA-TPGS nanoparticles. In vitro, cytotoxicity experiment showed advantages of the DMAB-modified PLGA-TPGS nanoparticle formulation over commercial Taxotere^®^ in terms of cytotoxicity against MCF-7 cells. In conclusion, oral chemotherapy by DMAB-modified PLGA-TPGS nanoparticle formulation is an attractive and promising treatment option for patients.

## Introduction

Oncology is one of the few areas of medicine where most patients are treated intravenously rather than receiving oral medications. Oral chemotherapy is attractive because of its convenience and ease of administration, particularly in a palliative setting. In addition, the oral route facilitates the use of more chronic treatment regimens, which result in prolonged exposure to anticancer drugs. However, most anticancer drugs such as Taxoids (paclitaxel and docetaxel) are not orally bioavailable, i.e., not absorbable in the gastrointestinal (GI) tract. This is because Taxoids have a very low level of oral bioavailability at less than 10% [1,2]. The low systemic exposure of Taxoids after oral drug administration is, at least in part, due to their high affinity for the multidrug efflux pump P-glycoprotein (P-gp) [3,4]. P-gp in the mucosa of the GI tract limits the absorption of the orally administered Taxoids and mediates their direct excretion into the gut lumen [3]. In addition, first-pass elimination by cytochrome P450 (CYP) isoenzymes in the liver and/or gut wall may also contribute to the low oral bioavailability of Taxoids [5,6]. Possible solutions for oral delivery of Taxoids and other anticancer drugs are currently under extensive investigation [2]. The general idea is to apply P-gp/P450 inhibitors such as cyclosporine to suppress the elimination process [7,8]. However, P-gp/P450 inhibitors may suppress the body's immune system and thus cause severe medical complications. Polymeric nanoparticles are of special interest from the pharmaceutical point of view. Polymeric nanoparticles could escape from the recognition of P-gp and thus bear the most potential to enhance the oral bioavailability of drugs that are otherwise poorly absorbed when administered orally [9-11]. Their submicron size and their large specific surface area favor their absorption compared to larger carrier. The nanoparticles could also shield incorporated drug molecules from the gastrointestinal tract (GIT) degradation as well as gut wall metabolism. In addition, the nanoparticles could bypass the liver and prevent the first-pass metabolism of the incorporated drug [12]. It has been fully accepted that nanoparticle surface properties are of outmost importance for their uptake by intestinal epithelial cells. Hence, many strategies have been developed to improve mucosal absorption of nanoparticles, either by modifying their surface properties or by coupling a targeting molecular at their surface [13]. In the present study, we proposed a novel nanoparticle formulation, i.e., biodegradable PLGA-TPGS nanoparticles modified with a cationic surfactant, didodecyldimethylammonium bromide (DMAB) (named DMAB/PLGA-TPGS NPs hereinafter), for oral chemotherapy using docetaxel as a therapeutic drug due to its excellent therapeutic effects against a wide spectrum of cancers and its commercial success as one of the top-selling anticancer agents.

Reports on the positive surface charge of DMAB provided the incentive to aid drug adsorption and delivery, since it is expected to ensure better interaction with the negatively charged cell membrane [14-16]. This can result in increased retention time at the cell surface, thus increasing the chances of particle uptake and improving oral drug bioavailability [17]. DMAB is capable of producing small and highly stable nanoparticles at 1% w/v concentration [18]. Due to the charged surface, the particle agglomeration is impeded. Thus, in this research, DMAB was absorbed on the nanoparticle surface by electrostatic attraction between positive and negative charges. In our design, the FDA-approved biodegradable polymer PLGA was employed to maintain the mechanical strength of the copolymer. d-α-tocopheryl polyethylene glycol 1,000 succinate (TPGS) is a water-soluble derivative of natural vitamin E, which is formed by esterification of vitamin E succinate with polyethylene glycol (PEG) 1,000. TPGS could improve drug permeability through cell membranes by inhibiting P-glycoprotein, and thus enhance absorption of drugs and reduce P-glycoprotein-mediated multidrug resistance in tumor cells [19-21]. It was found that TPGS could also effectively inhibit the growth of human lung carcinoma cells from in vitro cell culture and implanted in nude mice [22]. The superior anticancer efficacy of TPGS is associated with its increasing ability to induce apoptosis and not due to its increased cell uptake into cells [22-24]. Synergistic antitumor effects could be obtained by the use of combinations of vitamin E isomers or derivatives in the presence of other anticancer agents [23]. In addition, TPGS-emulsified nanoparticles have been shown higher drug encapsulation and cellular uptake, longer half-life and higher therapeutic effects of the formulated drug than those emulsified by poly (vinyl alcohol) (PVA), a widely used emulsifier in nanoparticle technology [21]. We were thus inspired to synthesize a novel biodegradable poly(lactide-co-glycolide)-d-a-tocopheryl polyethylene glycol 1,000 succinate (PLGA-TPGS) random copolymer for nanoparticle formulation of small molecule drug chemotherapy [21].

## Materials and Methods

### Materials

D,L-lactide (3,6-dimethyl-1,4-dioxane-2,5-dione, C6H8O4) with purity above 99% and didodecyldimethylammonium bromide (DMAB) were purchased from Sigma–Aldrich (St. Louis, MO, USA). d-α-tocopheryl polyethylene glycol 1,000 succinate (TPGS, C_33_O_5_H_54_ (CH_2_CH_2_O)_23_), PLGA (50:50, MW 50,000), glycolide (1,4-Dioxane-2,5-dione, C_4_H_4_O_4_), stannous octoate (Sn(OOCC_7_H_15_)_2_) and 3-(4,5-dimethylthiazol-2-yl)-2,5-diphenyltetrazolium bromide (MTT) were also supplied from Sigma–Aldrich (St. Louis, MO, USA). Docetaxel of purity 99.8% was purchased from Shanghai Jinhe Bio-Technology Co. Ltd (Shanghai, China). Acetonitrile and methanol were purchased from EM Science (ChromAR, HPLC grade, Mallinckrodt Baker, USA). All other chemicals used were of the highest quality commercially available. Ultrahigh pure water produced by Boon Environmental Tech. Industry Co., Ltd (Tianjin, China) was utilized throughout all experiments.

### Synthesis and Characterization of PLGA-TPGS Random Copolymers

PLGA-TPGS random copolymers were synthesized from lactide, glycolide and TPGS in the presence of stannous octoate as a catalyst via ring opening polymerization [21]. In brief, weighted amounts of lactide, glycolide, TPGS and 0.5 wt% stannous octoate (in distilled toluene) were added in a flask. The mixture was heated to 145°C and allowed to react for 12 h. Synthesis was carried out under an oxygen- and moisture-free environment. The product was dissolved in DCM and then precipitated in excess cold methanol to remove unreacted lactide monomers and TPGS. The final product was collected by filtration and vacuum dried at 45°C for 2 days. The TPGS content and number-averaged molecular weight of the copolymer were determined by 1H NMR in CDCl3 at 300 Hz (Bruker ACF300). The weight-averaged molecular weight and molecular weight distribution were determined by gel permeation chromatography (Waters GPC analysis system with RI-G1362A refractive index detector, Waters, Milford, USA).

### Preparation of DMAB-Modified Nanoparticles

Nanoparticles were fabricated by a solvent extraction/evaporation method with slight modifications [25,26]. Briefly, a given amount of docetaxel and 100 mg PLGA-TPGS copolymer were dissolved in 8 ml dichloromethane (DCM). The formed solution was poured into 120 ml of 0.03% (w/v) TPGS solution under gentle stirring. The mixture was sonicated for 120 s at 25 W output to form O/W emulsion. The emulsion was then evaporated overnight under reduced pressure to remove DCM. The particle suspension was centrifuged at 22,000 rpm for 20 min, and then washed three times to remove TPGS and unencapsulated drug. The resulted particles were resuspended in 10 ml DI water and freeze-dried. The surface modification of the PLGA-TPGS nanoparticles was carried out by a method described previously [14]. DMAB was dissolved in DI water at a concentration of 0.5 mg/ml. Preweighed nanoparticles were suspended in this solution at a concentration of 9.5 mg/ml by sonication at 25 W power output for 60 s over an ice bath, and then were collected by ultracentrifugation. In addition, the fluorescent coumarin-6-loaded nanoparticles were prepared in the same way, except 0.1% (w/v) coumarin-6 was encapsulated instead of docetaxel. DMAB-modified PLGA nanoparticles were prepared by the same method.

### Characterization of Nanoparticles

#### Size Analysis and Surface Charge

Size and size distribution of nanoparticles were determined by Dynamic Light Scattering (Zetasizer Nano ZS90, Malvern Instruments LTD., Malvern, UK). The particles (about 2 mg) were suspended in deionized water before measurement. Zeta potential of the nanoparticles was measured by Laser Doppler Anemometry (LDA; Zetasizer Nano ZS90, Malvern Instruments LTD., Malvern, UK). The measurement was performed triplicate.

#### Surface Morphology

The particle morphologies were examined by a field emission scanning electron microscopy (FESEM), using a JEOL JSM-6700F system operated at a 5.0 kV accelerating voltage. To prepare samples for FESEM, the particles were fixed on the stub by a double-sided sticky tape and then coated with platinum layer by JFC-1,300 automatic fine platinum coater (JEOL, Tokyo, Japan) for 40 s.

#### Drug Content and Entrapment Efficiency

Drug loading content and entrapment efficiency (EE) of the nanoparticles were determined by HPLC (LC 1200, Agilent Technologies, Santa Clara, CA) according to previously published methods [13,14]. Briefly,5 mg nanoparticles were dissolved in 1 ml DCM under vigorous vortexing. This solution was transferred to 5 ml of mobile phase consisting of deionized water and acetonitrile (50:50, v/v). A nitrogen stream was introduced to evaporate the DCM for about 15 min, and then a clear solution was obtained for HPLC analysis. A reverse-phase Inertsil^®^ C-18 column (150 mm × 4.6 mm, pore size 5 mm, GL science Inc, Tokyo, Japan) was used. The flow rate of mobile phase was 1 ml/min. The column effluent was detected at 227 nm with a UV/VIS detector. The drug EE was defined as the ratio between the amount of docetaxel encapsulated in the nanoparticles and that added in the process. Experiments were performed in triplicate, and results are expressed as mean ± standard deviation (SD).

#### In Vitro Drug Release

Fifteen milli-gram docetaxel-loaded nanoparticles were dispersed in 5 ml release medium (phosphate buffer solution of pH 7.4 containing 0.1% w/v Tween 80) to form a suspension. Tween 80 was used to increase the solubility of docetaxel in the buffer solution and avoid the binding of docetaxel to the tube wall. The suspension was transferred into a Regenerated Cellulose Dialysis Membrane (Spectra/Por 6, MWCO = 1,000, Spectrum, Houston, TX, USA). Then, the closed bag was put into a centrifuge tube and immersed in 15 ml release medium. The tube was put in an orbital water bath shaking at 120 rpm at 37.0°C. Ten milliliter of solutions were periodically removed for analysis and replaced with fresh medium. The collected samples were extracted with 2 ml DCM and reconstituted in 5 ml mobile phase. A nitrogen stream was introduced to evaporate the DCM. The analysis procedure was the same as for the measurement of encapsulation efficiency.

### Cellular Uptake of Nanoparticles

Caco-2 cells of passage 30–35 (American Type Culture Collection, VA) were used in this study to simulate the GI barrier for oral chemotherapy, which were grown in 25-cm^2^ tissue culture flasks maintained at 37°C in a humidified environment of 5% CO_2_. The medium, Dubelco's modified essential medium (DMEM, Sigma D1152) supplemented with 20% fetal bovine serum, 100 U/ml penicillin and 100 (g/ml streptomycin (Sigma) was freshened every 3 days. After 90% confluence, the cells were collected by 0.25% of Trypsin–EDTA solution (Sigma) and cultured in 96-well black plate (Costa^®^, Corning Incorporated) at a density of 1.3 × 104 cells/well; after the cells reached confluence, the cells were equilibrated with HBSS at 37°C for 1 h and then incubated with coumarin-6-loaded nanoparticle suspension. The nanoparticles were dispersed in the medium at concentration of 100, 250 and 500 (g/ml). The wells with nanoparticles were incubated at 37°C for 2 h. After incubation, the suspension was removed and the wells were washed three times with 50 μl cold PBS to eliminate traces of nanoparticles left in the wells. After that, 50 μl of 0.5% Triton X-100 in 0.2 N NaOH was introduced into each sample wells to lyse the cells. The fluorescence intensity of each sample well was measured by microplate reader (GENios, Tecan, Switzerland) with excitation wave length at 430 nm and emission wavelength at 485 nm. Cell uptake efficiency was expressed as the percentage of cells associated fluorescence versus the fluorescence present in the feed solution. Culture of human breast adenocarcinoma cell line MCF-7 cells (passage 30–35, American Type Culture Collection) and their uptake of the coumarin-6-loaded nanoparticles were performed in the same way.

Caco-2 cells were re-seeded in the chambered cover glass system (LABTEK^®^, Nagle Nunc, IL). After the cells were incubated with 250 μg/ml coumarin-6-loaded DMAB-modified PLGA-TPGS nanoparticle suspension at 37°C for 2 h, the cells were rinsed with cold PBS for three times and then fixed by ethanol for 20 min. The cells were further washed twice with PBS, and the nuclei were counterstained with propidium iodide (PI) for 30 min. The cell monolayer was washed twice with PBS and mounted in Dako^®^ fluorescent mounting medium (Dako, CA) to be observed by confocal laser scanning microscope (CLSM) (Zeiss LSM 410) with an imaging software, Fluoview FV500.

### In Vitro Cell Viability

MCF-7 cells were seeded in 96-well plates at the density of 5,000 viable cells per well and incubated 24 h to allow cell attachment. The cells were incubated with docetaxel-loaded PLGA-TPGS nanoparticle suspension, DMAB-modified PLGA-TPGS nanoparticle suspension and commercial Taxotere^®^ at 0.25, 2.5, 12.5 and 25 μg/ml equivalent docetaxel concentrations and drug-free DMAB-modified PLGA-TPGS nanoparticle suspension with the same amount of nanoparticles for 24, 48 and 72 h, respectively. At determined time, the formulations were replaced with DMEM containing MTT (5 mg/ml) and cells were then incubated for additional 4 h. MTT was aspirated off and DMSO was added to dissolve the formazan crystals. Absorbance was measured at 570 nm using a microplate reader (Bio-Rad Model 680, UK). Untreated cells were taken as control with 100% viability, and cells without addition of MTT were used as blank to calibrate the spectrophotometer to zero absorbance. IC_50_, the drug concentration at which inhibition of 50% cell growth was observed, in comparison with that of the control sample, was calculated by curve fitting of the cell viability data. Experiments were performed in triplicate and results are expressed as mean ± SD.

### Statistical Methodology

The results were expressed as mean ± SD. The significance of differences was assessed using Student's *t*-test and was termed significance when *P* < 0.05.

## Results and Discussions

### Characterization of PLGA-TPGS Random Copolymer

**Figure 1:**
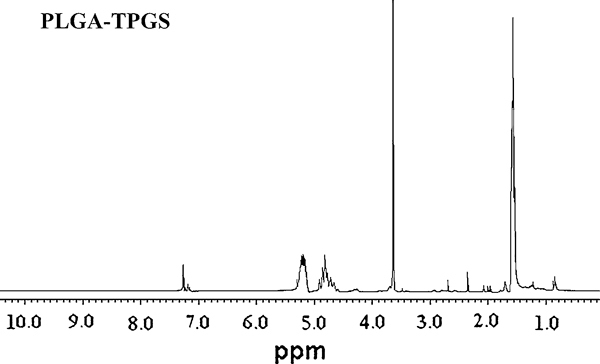
**Typical 1H-NMR spectra of PLGA-TPGS random copolymer**.

The chemical structure of the PLGA-TPGS random copolymer synthesized in our research can be found from our earlier work [21]. The Characterization of 1H NMR and GPC is tabulated in Table 1. The weight-averaged and number-averaged molecular weight of the PLGA-TPGS random copolymer with PLGA:TPGS = 90:10 were determined to be 28,530 and 21,944, respectively, with polydispersity of 1.30. As shown in Figure 1, the copolymer was successfully synthesized at the characteristic peak of 5.2 and 1.69 ppm for PLA, 4.82 ppm for PGA and at that of 3.65 ppm for TPGS, respectively.

**Table 1:** Characteristics of the PLGA-TPGS random copolymer

Copolymers	TPGS feed content (%)	**TPGS content**^**a**^**(%)**	**Molecular weight**^**b**^		
			
			PI (*Mw*/*Mn*)	*Mw*	*Mn*
PLGA-TPGS 90:10	15.00	10.44	1.30	28,530	21,944

^a^Calculated by 1H NMR

^b^Calculated by GPC

### Characterization of Drug-Loaded Nanoparticles

#### Size, Zeta Potential and Drug Entrapment Efficiency

The size and size distribution of the 5% DMAB-modified PLGA nanoparticles(ANP), unmodified PLGA-TPGS nanoparticles(BNP), 5% DMAB-modified PLGA-TPGS nanoparticles(CNP) and 20% DMAB-modified PLGA-TPGS nanoparticles(DNP) prepared in this research are shown in Table 2. The particle size is a key parameter used to determine the cellular uptake of the nanoparticles. The permeability of the particles through the intestinal mucosa decreases with increasing the particle size reaching a cut-off at around 500 nm [27,28]. The prepared nanoparticles were of 200–300 nm diameter, which is in the size range favoring the intestinal uptake of the nanoparticles [2]. The results also showed that the addition of DMAB resulted in a slight decrease in particle size. Zeta potential analysis confirmed that surface modification with 5% DMAB changed the PLGA-TPGS nanoparticles from a negative surface charge of -21.87 to a significantly positive charge of +32.23. Literature suggests that positive surface charge enhances mucosal uptake due to anionic nature of mucous layer [18]. It has been also reported that the efficiency of arterial uptake of nanoparticles could be improved by at least sevenfold after DMAB modification of nanoparticles [29].

**Table 2:** Effects of DMAB modification on size, entrapment efficiency and zeta potential

Group	Polymer	Size (nm)	PDI	Zeta Potential (mV)	Drug loading (%)	EE (%)	DMAB Modification (%)
ANP	PLGA	239.82 ± 8.64	0.299	-28.58 ± 4.44	8.93	88.26	5
BNP	PLGA-TPGS	253.51 ± 5.38	0.264	-21.87 ± 2.11	9.83	98.27	None
CNP	PLGA-TPGS	226.33 ± 3.56	0.251	32.23 ± 3.55	9.62	96.23	5
DNP	PLGA-TPGS	219.42 ± 5.24	0.199	34.15 ± 4.28	9.21	92.12	20

*PDI* polydispersity index, *EE* drug entrapment efficiency, *n* = 3

As the drug entrapment efficiency (EE) regards, it can be seen from Table 2 that the 5% DMAB-modified PLGA-TPGS nanoparticles (CNP) achieved much higher EE than the 5% DMAB-modified PLGA nanoparticles (ANP). This might be contributed to the self-emulsification effect of the PLGA-TPGS copolymer [2,21].

#### Surface Morphology

**Figure 2:**
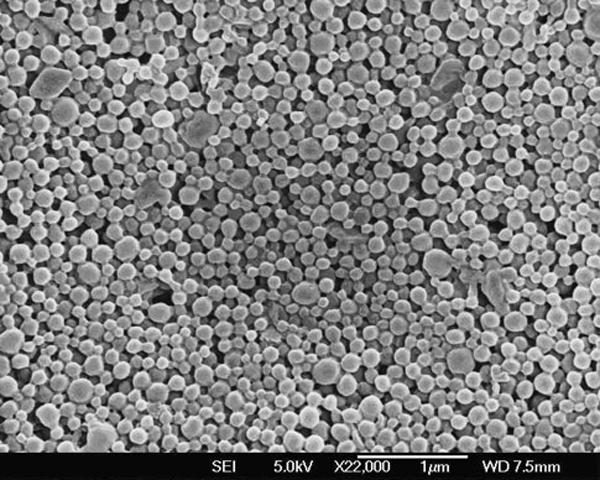
**FESEM image of docetaxel-loaded 5% DMAB-modified PLGA-TPGS nanoparticles**.

Surface morphology of the 5% DMAB-modified PLGA-TPGS nanoparticles (CNP) was examined by FESEM. Figure 2 shows the FESEM images of 5% DMAB-modified PLGA-TPGS nanoparticles (CNP). The FESEM image further confirmed the particle size detected from the DLS. The morphology of the nanoparticles formed was recorded as smooth and spherical in shape.

#### In vitro Drug Release

**Figure 3:**
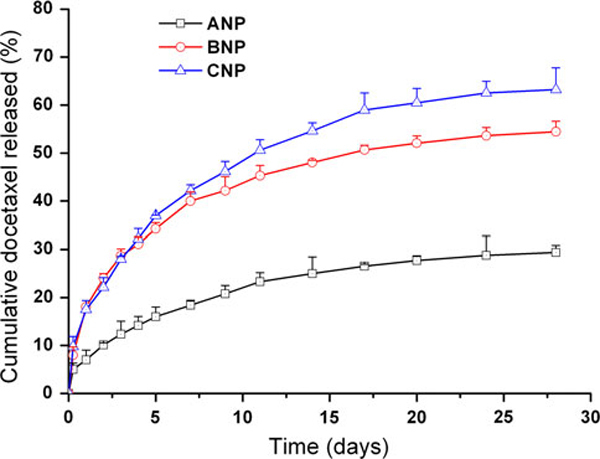
**The in vitro release profile of docetaxel-loaded 5% DMAB-modified PLGA nanoparticles (ANP), unmodified PLGA-TPGS nanoparticles (BNP) and 5% DMAB-modified PLGA-TPGS nanoparticles (CNP)**.

The in vitro drug release profiles of the 5% DMAB-modified PLGA nanoparticles (ANP),unmodified PLGA-TPGS nanoparticles (BNP) and 5% DMAB-modified PLGA-TPGS nanoparticles (CNP) in the first 28 days are shown in Figure 3. The drug release from the 5% DMAB-modified PLGA-TPGS nanoparticles (CNP) was found to be 36.98% and 63.22% of the encapsulated drug in the first 5 days and after 28 days, respectively, which was much faster than the 5% DMAB-modified PLGA nanoparticles (ANP), which is only 15.99% and 29.39%, respectively, in the same periods. The faster drug release of 5% DMAB-modified PLGA-TPGS nanoparticles (CNP) may be attributed to the lower molecular weight and the higher hydrophilicity of PLGA-TPGS copolymer in comparison with the PLGA nanoparticles. It causes the copolymer to swell and to degrade faster, thus promoting the drug release from the nanoparticles. It can also be seen from Figure 3 that drug release from the 5% DMAB-modified PLGA-TPGS nanoparticles (CNP) was slightly faster than that of unmodified PLGA-TPGS nanoparticles (BNP). Such a phenomenon may be attributed to slightly smaller particle size of 5% DMAB-modified PLGA-TPGS nanoparticles (CNP). It may be thought that in vitro, drug release should be evaluated ideally in a release medium which can better simulate the acidic condition of the gastrointestinal fluid. However, this is not an important issue since the nanoparticles would stay with the GI track for a few hours only. Drug release in plasma and in the cancer cells plays a more important role.

### Uptake of Coumarin-6-Loaded Nanoparticles by Caco-2 and MCF-7 Cells

**Figure 4:**
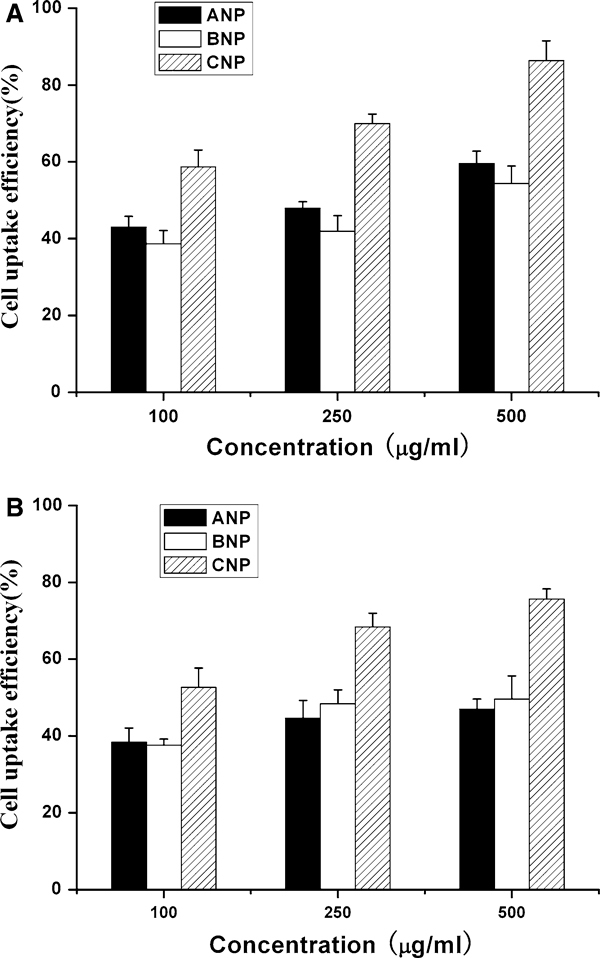
**Cellular uptake of coumarin-6-loaded 5% DMAB-modified PLGA nanoparticles (ANP), unmodified PLGA-TPGS nanoparticles (BNP) and 5% DMAB-modified PLGA-TPGS nanoparticles (CNP) by a Caco-2 and b MCF-7 cells after 2-h incubation**.

Caco-2 cells are a widely accepted model to predict permeability and absorption of compounds in humans [30]. Taxoids have been extensively used to treat metastatic breast cancer. The fluorescence uptake by the MCF-7 cells could provide a useful model to assess the in vitro therapeutic effect of the Taxoids in the various formulations for breast cancer treatment [31,32]. The cellular uptake of coumarin-6-loaded 5% DMAB-modified PLGA nanoparticles (ANP), unmodified PLGA-TPGS nanoparticles (BNP) and 5% DMAB-modified PLGA-TPGS nanoparticles (CNP) was thus evaluated in this research using Caco-2 cell line as in vitro model of the GI barrier and MCF-7 cell line as model cancer cells. The cellular uptake efficiency of the coumarin-6-loaded nanoparticles by Caco-2 and MCF-7 cells was assayed upon 2-h incubation, and the results are shown in Figure 4.

It can be observed from Figure 4a that there is an increasing trend in the Caco-2 cellular uptake which shows the 5% DMAB-modified PLGA-TPGS nanoparticles (CNP) >5% DMAB-modified PLGA nanoparticles (ANP) >unmodified PLGA-TPGS nanoparticles (BNP). Such advantages are particle concentration dependent. The 5% DMAB-modified PLGA-TPGS nanoparticles (CNP) resulted in 1.37-, 1.46- and 1.45-fold higher cellular uptake than that of 5% DMAB-modified PLGA nanoparticles (ANP), and 1.52-, 1.67- and 1.59-fold higher cellular uptake than that of unmodified PLGA-TPGS nanoparticles (BNP) at the incubated particle concentration of 100, 250 and 500 μg/ml, respectively.

Figure 4b shows that the cellular uptake efficiency of the coumarin-6-loaded DMAB-modified PLGA-TPGS nanoparticles (CNP) by MCF-7 cells is higher than that of 5% DMAB-modified PLGA nanoparticles (ANP) and unmodified PLGA-TPGS nanoparticles (BNP), which is also found dose-dependent. The 5% DMAB-modified PLGA-TPGS nanoparticles (CNP) resulted in 1.37-, 1.53- and 1.61-fold higher cellular uptake than that of 5% DMAB-modified PLGA nanoparticles (ANP), and 1.40-, 1.41- and 1.52-fold higher cellular uptake than that of unmodified PLGA-TPGS nanoparticles (BNP) at the incubated particle concentration of 100, 250 and 500 μg/ml, respectively. The positive surface charge of DMAB provided the incentive to aid drug delivery, since it is expected to ensure better interaction with the negatively charged cell membrane [14-16]. This resulted in increased retention time at the cell surface, thus increasing the chances of particle uptake and improving oral drug bioavailability [17].

**Figure 5:**
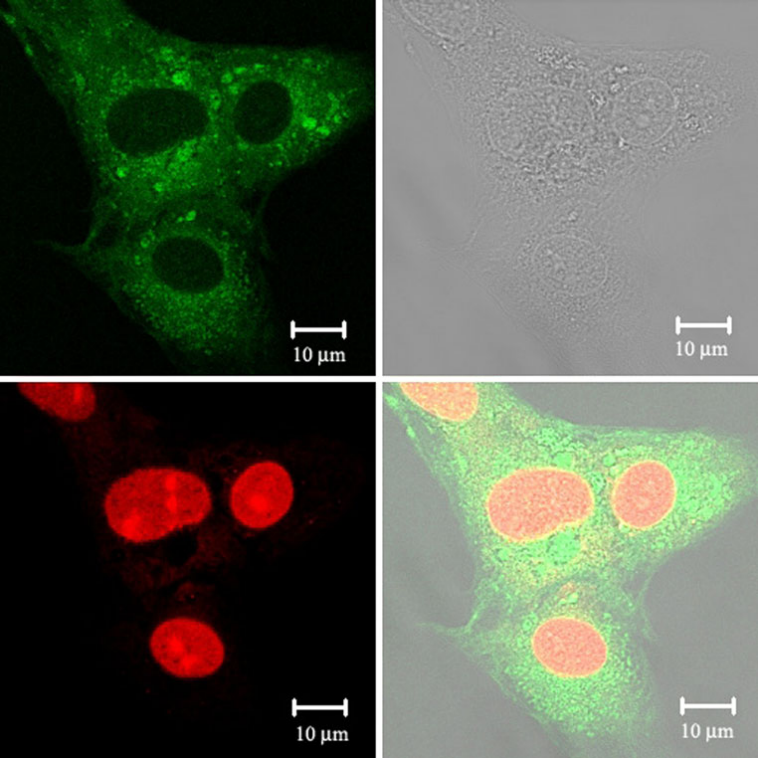
**Confocal laser scanning microscopy (CLSM) images of HeLa cells after 2 h incubation with coumarin-6-loaded5% DMAB-modified PLGA-TPGS nanoparticles at 37.0°C**. The cells were stained by propidium iodide (*red*) and the coumarin-6-loaded nanoparticles are *green*. The cellular uptake is visualized by overlaying images obtained by *white* light, FITC filter and PI filter: *upper-left image* from FITC channel; *upper-right image* from transmitted light channel; *lower-left image* from PI channel; *lower-right image* from combined transmitted light channel, PI channel and FITC channel.

Figure 5 shows confocal laser scanning microscopy (CLSM) images of Caco two cells after 2 h incubation with the coumarin-6-loaded 5% DMAB-modified PLGA-TPGS nanoparticles at 250 μg/ml nanoparticle concentration, in which, the upper-left image was obtained from FITC channel (green), the lower-left one was from propidium iodide (PI) channel (red), the upper-right image was from transmitted light channel (black and white), and the lower-right image was the combination of all the three images. It can be seen from this figure that the fluorescence of the coumarin-6-loaded 5% DMAB-modified PLGA-TPGS nanoparticles (green) is located in the cytoplasm around the nucleus (red, stained by PI), indicating the nanoparticles has been internalized into the cells [33].

### Cell Viability

**Figure 6:**
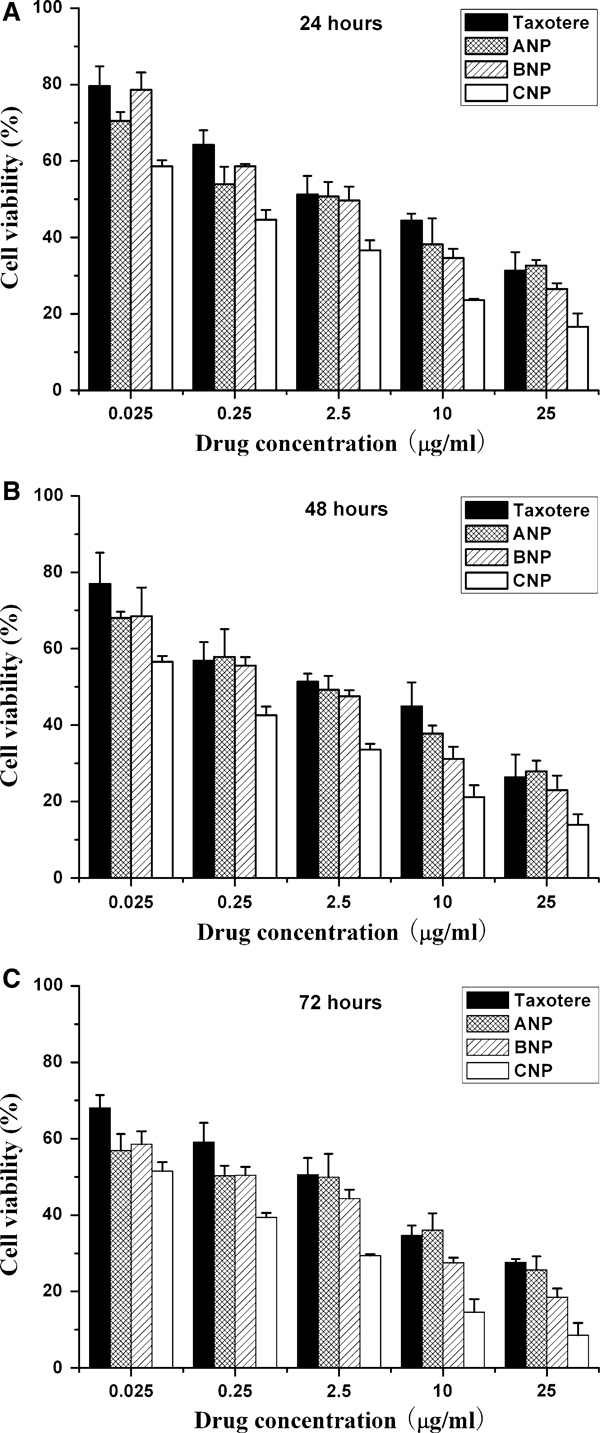
**Viability of MCF-7 cells cultured with docetaxel-loaded 5% DMAB-modified PLGA nanoparticles (ANP), unmodified PLGA-TPGS nanoparticles (BNP) and 5% DMAB-modified PLGA-TPGS nanoparticles (CNP) in comparison with that of Taxotere^®^ at the same docetaxel dose (*n* = 6)**.

Figure 6 shows the viability of MCF-7 cancer cells after 24 (upper), 48 (middle) and 72 (lower) hour cell culture with docetaxel formulated in the 5% DMAB-modified PLGA nanoparticles (ANP), unmodified PLGA-TPGS nanoparticles (BNP) and 5% DMAB-modified PLGA-TPGS nanoparticles (CNP) respectively in comparison with that of the Taxotere^®^ formulation at the same 0.025, 0.25, 2.5, 10 and 25 μg/ml docetaxel dose (*n* = 6). It can be concluded from this figure that in general (1) All 3 nanoparticle formulations showed advantages in decreasing the cancer cell viability (i.e. increasing the cancer cell mortality) versus the current clinical dosage form Taxotere^®^ and the 5% DMAB-modified PLGA-TPGS nanoparticles (CNP) can have even better effects than unmodified PLGA-TPGS nanoparticles (BNP). Such advantages of the nanoparticle formulations can be contributed to the effects of TPGS and DMAB component of the nanoparticles in enhancing cellular uptake of the nanoparticles. (2) The advantages in cancer cell viability of the 5% DMAB-modified PLGA-TPGS nanoparticles (CNP) >the unmodified PLGA-TPGS nanoparticles (BNP) >the Taxotere^®^ formulation is dependent on the incubation time. This may be contributed to the controlled release manner of the nanoparticle formulation. (3) The advantages in cancer cell viability of the 5% DMAB-modified PLGA-TPGS nanoparticles (CNP) >the unmodified PLGA-TPGS nanoparticles (BNP) >the Taxotere^®^ formulation is also dependent on the drug concentration. The higher the drug concentration, the more significant effects would be obtained.

The advantages in cancer cell viability of the 5% DMAB-modified PLGA-TPGS nanoparticles (CNP) >the unmodified PLGA-TPGS nanoparticles (BNP) >the Taxotere^®^ formulation can be quantitatively analyzed by IC_50_, which is defined as the drug concentration at which 50% of the cells in culture have been killed in a designated time period. Table 3 gives IC_50_ of MCF-7 cells after 24-, 48-, 72-h incubation with docetaxel formulated in the Taxotere^®^, 5% DMAB-modified PLGA nanoparticles (ANP), unmodified PLGA-TPGS nanoparticles (BNP) and 5% DMAB-modified PLGA-TPGS nanoparticles (CNP), respectively, which are obtained from Figure 6. The results showed that the IC_50_ value for MCF-7 cells was decreased from 2.610, 1.640 and 0.911 to 0.121, 0.088 and 0.054 μg/ml for 5% DMAB-modified PLGA-TPGS nanoparticle formulations (CNP) after 24-, 48- and 72-h incubation, respectively. As time goes by, the 5% DMAB-modified PLGA-TPGS nanoparticle formulation (CNP) showed better and better in vitro therapeutic effects for MCF-7 cells than commercial Taxotere^®^. This is because the accumulative drug release was only 17.48, 22.15 and 27.98% for 5% DMAB-modified PLGA-TPGS nanoparticle formulation (CNP) after 24, 48 and 72 h (Figure 3), respectively, and the release started from 0% while the Taxotere^®^ immediately became 100% available for the MCF-7 cells in culture. Furthermore, the degradation of PLGA-TPGS random copolymer may release the TPGS components, which have synergistic anticancer activity in the presence of anticancer agent [23,24], thus increasing cancer cell mortality.

**Table 3:** IC_50_ of MCF-7 cells after 24-, 48-, 72-h incubation with docetaxel formulated in the Taxotere^®^, 5% DMAB-modified PLGA nanoparticles (ANP), unmodified PLGA-TPGS nanoparticles (BNP) and 5% DMAB-modified PLGA-TPGS nanoparticles (CNP)

Incubation time (*h*)	**IC**_**50**_**(μg/ml)**			
	
	ANP	BNP	CNP	**Taxotere**^**®**^
24	1.144	1.300	0.121	2.610
48	0.926	0.590	0.088	1.640
72	0.272	0.204	0.054	0.911

## Conclusion

We developed three types of nanoparticle formulation from biodegradable PLGA-TPGS random copolymer for oral administration of anticancer drugs with docetaxel employed as a model drug, which include 5% DMAB-modified PLGA nanoparticles (ANP), unmodified PLGA-TPGS nanoparticles (BNP) and 5% DMAB-modified PLGA-TPGS nanoparticles (CNP). The design of the nanoparticle matrix material was made to take advantages of TPGS in nanoparticle preparation technology such as high emulsification effects and high drug entrapment efficiency, enhancement of therapeutic effects such as reducing P-glycoprotein-mediated multidrug resistance and superior anticancer efficacy as well as those in drug formulation such as high cellular adhesion and adsorption. DMAB was used to increase retention time at the cell surface, thus increasing the chances of particle uptake and improving oral drug bioavailability. The results showed that the DMAB-modified PLGA-TPGS nanoparticles have significantly higher level of the cellular uptake than that of DMAB-modified PLGA nanoparticles and unmodified PLGA-TPGS nanoparticles. In vitro, cytotoxicity experiment showed advantages of the DMAB-modified PLGA-TPGS nanoparticle formulation over commercial Taxotere^®^ in terms of cytotoxicity against MCF-7 cells. In conclusion, oral chemotherapy by DMAB-modified PLGA-TPGS nanoparticle formulation is an attractive and promising treatment option for patients.
